# Norepinephrine Regulation of Ventromedial Hypothalamic Nucleus Astrocyte Glycogen Metabolism

**DOI:** 10.3390/ijms22020759

**Published:** 2021-01-13

**Authors:** Karen P. Briski, Mostafa M. H. Ibrahim, A. S. M. Hasan Mahmood, Ayed A. Alshamrani

**Affiliations:** School of Basic Pharmaceutical and Toxicological Sciences, College of Pharmacy, University of Louisiana Monroe, Monroe, LA 71201, USA; ibrahimm@warhawks.ulm.edu (M.M.H.I.); mahmooas@warhawks.ulm.edu (A.S.M.H.M.); alshamaa@warhawks.ulm.edu (A.A.A.)

**Keywords:** ventromedial hypothalamic nucleus, norepinephrine, adrenergic receptor, glycogen phosphorylase brain type, laser-catapult microdissection

## Abstract

The catecholamine norepinephrine (NE) links hindbrain metabolic-sensory neurons with key glucostatic control structures in the brain, including the ventromedial hypothalamic nucleus (VMN). In the brain, the glycogen reserve is maintained within the astrocyte cell compartment as an alternative energy source to blood-derived glucose. VMN astrocytes are direct targets for metabolic stimulus-driven noradrenergic signaling due to their adrenergic receptor expression (AR). The current review discusses recent affirmative evidence that neuro-metabolic stability in the VMN may be shaped by NE influence on astrocyte glycogen metabolism and glycogen-derived substrate fuel supply. Noradrenergic modulation of estrogen receptor (ER) control of VMN glycogen phosphorylase (GP) isoform expression supports the interaction of catecholamine and estradiol signals in shaping the physiological stimulus-specific control of astrocyte glycogen mobilization. Sex-dimorphic NE control of glycogen synthase and GP brain versus muscle type proteins may be due, in part, to the dissimilar noradrenergic governance of astrocyte AR and ER variant profiles in males versus females. Forthcoming advances in the understanding of the molecular mechanistic framework for catecholamine stimulus integration with other regulatory inputs to VMN astrocytes will undoubtedly reveal useful new molecular targets in each sex for glycogen mediated defense of neuronal metabolic equilibrium during neuro-glucopenia.

## 1. Overview

The brain consumes a disproportionate fraction of bodily energy relative to its size in order to maintain neuron plasma membrane electrical stability and synaptic firing. Inadequate access to the principal substrate fuel glucose from the systemic circulation jeopardizes these vital brain cell functions, potentially leading to neurological dysfunction or nerve cell injury. The brain is continuously appraised of cellular metabolic equilibrium by dedicated interoceptive cells located within and outside the central nervous system. Input from these metabolic sensors is utilized by a distinctive neural glucostatic circuitry to regulate autonomic, neuroendocrine and behavioral outflow that maintains plasma glucose within a narrow physiological range. The final common control of these diverse functions derives from the hypothalamus, the principal autonomic motor center in the brain. The ventromedial hypothalamic nucleus (VMN), a critical component of the glucostatic network, functions to integrate multiple metabolic cues including nutrient profiles and indicators of peripheral (adipose tissue) energy stores to shape glucose counter-regulation. The complex carbohydrate glycogen is stored in brain astrocytes as a crucial metabolic fuel depot [1,2,3]. An emerging research topic of interest is the potential impact of glycogen metabolism on the VMN gluco-regulatory function. Discussed here is recent evidence that documented effects of the catecholamine transmitter norepinephrine (NE) on VMN metabolic signaling may involve, in part, governance of physiological stimulus-specific cues for glycogen mobilization.

## 2. Neural Regulation of Glucostasis

Glucose homeostasis results from the stabilization of plasma glucose levels within the relatively limited range of approximately 3.9–8.3 mmol/L despite dynamic variations in glucose acquisition by dietary delivery/endogenous production versus metabolism [4]. Plasma glucose decrements trigger a series of responses that include the inhibition of insulin secretion and induction at various glycemic thresholds of neurogenic signs (tremor, palpitations, sweating, hunger, etc.) and the secretion of synergistic counter-regulatory hormones (e.g., glucagon, epinephrine, cortisol and growth hormones) that elevate glucose by stimulating liver glycogenolysis and gluconeogenesis and limiting peripheral glucose utilization. Gluco-regulation is implemented by an expansive network of peripheral and central nervous system components, which processes signals on cellular and systemic energy stability to control autonomic and neuroendocrine motor outflow [5]. Specialized metabolic-sensory interoceptive neurons located in distinct hypothalamic (arcuate hypothalamic nucleus, VMN, lateral hypothalamic area) and hindbrain (dorsal vagal complex; DVC) structures provide a dynamic cellular energy readout via augmentation (‘glucose-inhibited’; GI neurons) or suppression (‘glucose-excited’; GE neurons) of their synaptic firing rate in response to the diminution of ambient substrate fuel levels [6,7,8,9,10,11]. 

## 3. Hindbrain-to-Hypothalamic Signaling in Gluco-Regulation

Neurons that exhibit electrical sensitivity to substrate fuel availability in vitro and in vivo reside in neuroanatomically distinct sensory (nucleus of the solitary tract, NTS; the principal visceral sensory nucleus in the brain), motor (dorsal motor nucleus vagus nerve) and circumventricular (area postrema) divisions of the DVC [7,8,12,13]. These cells, unlike electrically unresponsive neurons in the DVC, express the low-affinity, high Km hexokinase glucokinase (GCK) [14], a characterized metabolic-sensory marker [15]. DVC-derived metabolic signals contribute to gluco-regulation as caudal hindbrain glucose anti-metabolite or monocarboxylate transporter (MCT) inhibitor administration elevates blood glucose levels [16,17], whereas the infusion of the oxidizable glycolytic metabolite L-lactate to the caudal hindbrain exacerbates insulin-induced hypoglycemia [17]. Electrophysiological mapping of the DVC shows that energy fuel-sensitive neurons occur predominantly within the caudal, e.g., visceral, NTS [8]. A2 noradrenergic neurons reside within the caudal NTS. Acquisition of these cells from that location by in situ immunocytochemistry/laser-catapult microdissection for single-cell qPCR gene expression analysis provided novel evidence for expression of hypoglycemia-sensitive GCK mRNA in these cells [18]. A2 neurons are functionally responsive to neuro-glucoprivation as they exhibit up-regulated Fos protein immunostaining [19] and dopamine-β-hydroxylase (DβH) mRNA expression [20] in response to glucose anti-metabolite administration. Lactate availability is a key monitored metabolic variable in the hindbrain as reductions in local MCT function stimulate Fos immuno-expression in key hypothalamic metabolic structures [21]. Hypoglycemia-induced gluco-regulatory cues likely originate from A2 neurons as lactoprivation resulting from systemic glucoprivation increases hypothalamic NE content while activating the ultra-sensitive energy gauge adenosine 5′-monophosphate-activated protein kinase (AMPK) in these but not other hindbrain catecholaminergic cell groups [22].

## 4. Astrocyte Involvement in Neuro-Metabolic Stability

Blood glucose gains primary access to the brain by constitutive glucose transporter (GLUT)-1 and GLUT-3 mediated uptake into astrocytes; specifically, end-feet processes of these glial cells, which function as an important component of the blood-brain barrier [23]. In the astrocyte cell compartment, glucose is converted to glucose-6-phosphate (Glc-6-P) by hexokinase enzyme action, and is then either processed through the glycolytic pathway to produce the oxidizable substrate fuel L-lactate or incorporated into the multi-branched polysaccharide glycogen [24]. Lactate is transferred between astrocytes and neurons by cell type-specific MCT-1 and -2 functions [25], respectively, to support nerve cell oxidative respiration and energy production [26].

## 5. Brain Glycogen Metabolism

In the brain, glycogen is maintained primarily within astrocytes [27,28,29]. Glycogen metabolism is controlled by antagonistic actions of glycogen synthase (GS) and glycogen phosphorylase (GP) enzymes, which correspondingly catalyze glycogen synthesis or breakdown. Reversible phosphorylation of GS and GP results in enzyme deactivation or activation, respectively. Several neurotransmitters including NE are implicated in the regulation of cortical astrocyte glycogen metabolism [30,31,32,33]. Brain glycogen mass is characterized by active turnover during normal brain activity and metabolic stability. A significant proportion of glucose acquired by astrocytes enters the ‘glycogen shunt’, which entails the sequential incorporation into and release of glucose from glycogen prior to entry into the glycolytic pathway for processing to lactate [34,35]. Glycogen synthesis confers long-term energy stability as it contributes to the sole storage form of glucose, wherein a maximal number of glucosyl units is assembled in the smallest possible volume with minimal impact on osmolarity, and provides a rapid, cytosolic source of substrate for ATP production [36]. The rate of glucose utilization in the brain is determined principally by hexokinase activity, which is subject to stringent feedback control by Glc-6-P. During rapid escalation of energy demand, glycogen is a primary source of Glc-6-P as it is capable of generating this molecule at a rate that exceeds that associated with glucose uptake and phosphorylation [33]. Brain glycogenolysis is augmented in response to the disequilibrium of energy supply/demand (e.g., during seizure, sleep deprivation or hypoglycemia [37,38]) to liberate glucosyl units for lactate generation [1,39]. Brain GP muscle type (GPmm) and brain type (GPbb) enzyme isoforms exhibit dissimilar cell type localization and regulation by phosphorylation versus AMP [40]. GPbb protein is expressed mainly in astrocytes with a small fraction also present in neurons, whereas GPmm occurs in astrocytes only. Phosphorylation causes complete GPmm or partial GPbb activation of these variants, while GPbb exhibits a higher affinity for and sensitivity to AMP activation than GPmm and requires AMP binding for optimal enzyme function and Km. Moreover, the GPbb, but not GPmm isoform is sensitive to reactive oxygen species [41,42]. Cortical astrocytes display up-regulated GPmm protein expression as a consequence of NE exposure, but enhanced GPbb profiles during energy deficiency in vitro [43].

## 6. Effects of Pharmacological Inhibition of VMN GP Activity on Metabolic Transmitter Signaling

Site-targeted delivery of the GP inhibitor 1,4-dideoxy-1,4-imino-d-Arabinitol (DAB) to the VMN augments local expression of neuronal nitric oxide synthase (nNOS), a marker protein for the gluco-stimulatory gaseous transmitter nitric oxide [44]. Meanwhile, inhibitory effects of this treatment paradigm on VMN glutamate decarboxylase_65/67_ (GAD) protein content infer that the diminution of local glycogen disassembly may suppress the gluco-inhibitory neurotransmitter γ-aminobutyric acid (GABA) [45]. In light of evidence that ventromedial hypothalamic lactoprivation stimulates counter-regulatory hormone release [46] by down-regulating GABA release [47], outcomes of the DAB treatment paradigm described above infer that astrocyte glycogen-derived substrate fuel stream is a critically monitored metabolic variable that shapes VMN metabolic neurochemical signaling. 

## 7. Hindbrain Noradrenergic Regulation of VMN Glycogen Metabolism

DVC NE governs hypoglycemic patterns of VMN AMPK activation and neurotransmitter marker protein expression [48]. Noradrenergic regulation of VMN glycogen metabolic enzyme protein expression was initially documented by studies that showed that exogenous NE inhibits GS protein expression but imposes divergent, e.g., stimulatory versus inhibitory, effects on AMP-sensitive GPbb and NE-sensitive GPmm profiles, respectively [49]. As VMN astrocytes obtained by laser-catapult microdissection express alpha_1_ (α_1_), alpha_2_- (α_2_) and beta_1_- (β_1_) adrenergic receptor (AR) proteins [50], it is reasonable to presume that the noradrenergic regulation of the GP variant expression involves, in part, direct input to these cells. Hindbrain lactoprivation is a potent physiological stimulus for amplified noradrenergic signaling to the hypothalamus [22]. In an effort to clarify whether endogenous hindbrain noradrenergic transmission regulates these VMN protein targets, studies were undertaken in our laboratory to investigate the effects of caudal hindbrain delivery of the MCT inhibitor α-cyano-4-hydroxycinnamic acid (4CIN) on VMN glycogen metabolic enzyme profiles [51]. Outcomes of that work revealed that 4CIN treatment significantly inhibited VMN GPmm levels, but at the same time increased tissue GPbb content. These findings imply that hindbrain sensor construal of diminished lactate provision as a manifestation of systemic energy insufficiency may facilitate VMN glycogenolysis under such circumstances through augmentation of expression of the AMP-sensitive GP isoform. 

**Figure 1:**
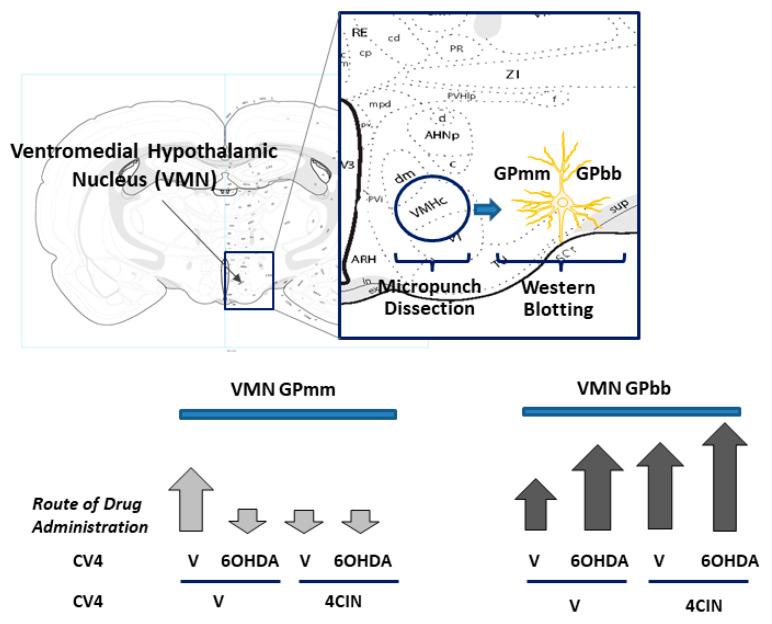
Differential hindbrain noradrenergic regulation of the ventromedial hypothalamic nucleus (VMN) glycogen phosphorylase brain (GPbb) versus muscle (GPmm) type protein expression. The coronal brain section illustration at the top depicts the location of the VMN within the mediobasal hypothalamus; that area is enlarged in the blue rectangle to show micropunch tool positioning for the collection of VMN tissue for the Western blot analysis of GPmm and GPbb proteins. The hollow circular punch tool was centered on the longitudinal axis of the VMN to acquire tissue from dorsomedial (dm), central (c) and ventrolateral regions of this nucleus. In the brain, GP proteins are expressed primarily in astrocytes but neurons also contain GPbb, albeit at relatively lower levels. Male rats were pretreated by the administration of vehicle (V) or the catecholamine neurotoxin 6-hydroxydopamine (6OHDA) into the caudal fourth ventricle (CV4) prior to the delivery of the monocarboxylate transporter inhibitor 4-alpha-cyano-cinnamic acid (4CIN). At the bottom, the arrows indicate the direction of various treatment effects on GPmm (left) and GPbb (right) expression relative to V-treated controls.

Further work utilized the selective catecholamine neurotoxin 6-hydroxydopamine (6OHDA) to address the role of noradrenergic transmission in the hindbrain lactoprivic regulation of the VMN GP variant expression [52]. Results showed that the stimulatory effects of 4CIN on DVC levels of the catecholamine biosynthetic enzyme dopamine-beta-hydroxylase, VMN NE content, and circulating glucose and counter-regulatory hormone levels were reversible by 6OHDA. Interestingly, 6OHDA suppressed or increased VMN GPmm and GPbb content, respectively, in non-4CIN-injected rats yet prevented the 4CIN inhibition of GPmm (Figure 1). Thus, homeostatic patterns of NE transmission evidently prioritize VMN glycogenolysis by NE stimulation versus AMP accumulation. Yet, signals of hindbrain metabolic instability are able to switch primary control of glycogen breakdown to AMP. Opposing effects of lactoprivic signaling on VMN GPmm versus GPbb protein expression likely facilitate energy deficit- rather than neurotransmitter-mediated glycogen breakdown under conditions of metabolic imbalance. At the same time, 6OHDA elevated basal VMN GAD expression but abolished 4CIN stimulation of this transmitter marker protein. The central finding of this work is that NE determines stimulus-specific VMN glycogen mobilization in accordance with metabolic state. Moreover, outcomes affirm the hindbrain lactoprivic regulation of glucostasis and identify NE involvement in key neural and endocrine responses to this hindbrain-derived cue. Further effort is required to determine if bi-directional, NE-dependent effects of hindbrain lactoprivation on GP and GAD expression involve a cause-and-effect relationship between these endpoints.

It should be noted that outcomes documenting lactoprivic augmentation of the gluco-inhibitory transmitter GABA likely reflect, to some extent, differences associated with this experimental model (which involves selective pharmacological activation of a single metabolic-sensory cell population, e.g., hindbrain A2 neurons) compared with systemic insulin-induced hypoglycemia (which is characterized by body-wide sensor activation). It is possible that under the latter circumstances, noradrenergic stimulation of GAD expression may be attenuated by a neurotransmitter cue(s), as-yet-unidentified, that is provided to the VMN by non-hindbrain energy sensors. Alternatively, up-regulated GABA transmission may be a signal of glycogen conservation due to negligible disassembly caused by down-regulated expression of NE-sensitive GPbb, alongside an absence of metabolic deficit activation of GPbb activity. These findings are consistent with earlier reports that NE depletion reduces glycogenolysis during energy imbalance [53].

## 8. NE Signaling Appraises the VMN of Hindbrain Glycogen Metabolic Status

In light of proof that ample tissue glycogen levels are maintained in the brainstem [54], it was relevant to examine the premise that hindbrain glycogen-derived fuel supply affects A2 energy stability and the governance of VMN metabolic transmitter signaling and glycogen metabolism [55]. Rats treated by DAB administration to the caudal fourth ventricle exhibited exogenous lactate-revocable changes in laser-catapult microdissected metabolic-sensory A2 noradrenergic neuron AMPK expression and VMN NE content. The ability of lactate to prevent DAB suppression of A2 nerve cell estrogen receptor-alpha (ERα) and G-protein-coupled estrogen receptor-1 (GPER) protein content advances the novel notion that glycogen-derived energy fuel supply may control estradiol input to these neurons. The latter findings align with reports that hindbrain ERα and ER-beta (ERβ) regulate hypoglycemic patterns of the VMN transmitter and glycogen metabolic protein expression albeit differently in each sex [56,57]. Interestingly, we found that lactate abolished the DAB up-regulation of the VMN GPbb content but did not modify the drug effects on GPmm protein profiles. Moreover, lactate repletion was found to reverse DAB augmentation of VMN NO and suppression of VMN GAD levels. The outcomes of this research affirm noradrenergic participation in the transmittal of cues on the hindbrain glycogen metabolism status to the VMN, and provide novel evidence that decreased glycogen disassembly and the resulting decline in oxidizable substrate fuel provision to hindbrain neurons is a stimulus for VMN glycogen mobilization and transmitter signals of metabolic deficiency.

## 9. Concentration-Dependent Noradrenergic Regulation of Hypothalamic Astrocyte Glycogen Metabolic Enzyme and Adrenergic Receptor Expression in Male Versus Female Primary Astrocyte Cultures

**Figure 2:**
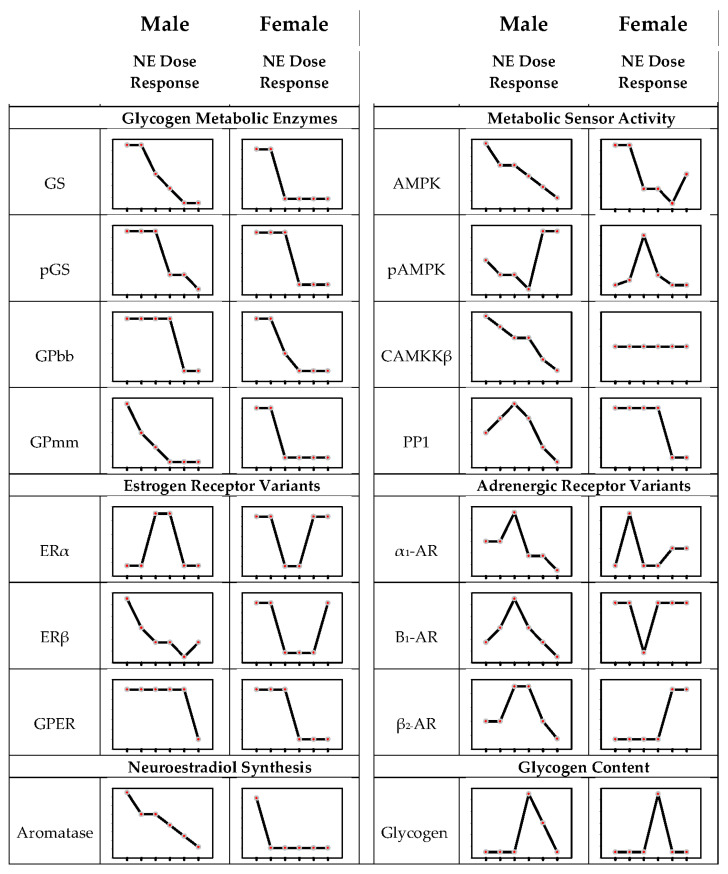
Norepinephrine (NE) regulation of the hypothalamic astrocyte glycogen metabolic enzyme and adrenergic and estrogen receptor variant protein expression and glycogen content in male versus female primary hypothalamic astrocyte cell cultures. In each graph, tick marks along the *x* axis indicate, from right to left, astrocyte incubation with NE over a dosage range of 0, 0.1, 1.0, 10.0, 100.0 or 1000 nM. Abbreviations: α_1_-AR: alpha_1_-adrenergic receptor; α_2_-AR: alpha_2_-adrenergic receptor; β_1_-AR: beta_1_-adrenergic receptor; β_2_-AR: beta_2_-adrenergic receptor; AMPK: 5′-AMP-activated protein kinase; CaMMKB: calcium/calmodulin-dependent protein kinase kinase-β; ERα: estrogen receptor-alpha; ERβ: estrogen receptor-beta; GS: glycogen synthase; GPbb: glycogen phosphorylase brain type; GPER: G-protein-coupled estrogen receptor-1; GPmm: glycogen phosphorylase muscle type; pAMPK: phosphoAMPK; pGS: phosphoGS; PP1: protein phosphatase-1.

The seminal finding that variation in the noradrenergic signal volume, governed by the hindbrain metabolic state, imposes a differential regulation of VMN glycogen metabolic enzyme expression prompts a consideration of molecular mechanisms that may control loss-or-gain or shift in direction of NE effects on these protein targets. In recent work, we used a hypothalamic astrocyte primary cell culture model [58] to address the premise that dosage-contingent NE effects on astrocyte GS and GP isoform protein expression and glycogen accumulation may correlate with differential patterns of the AR variant expression. Modes of hindbrain noradrenergic signaling of metabolic deficiency evidently differed between sexes, as systemic hypoglycemia increased the VMN NE content in males [56] but diminished these levels in the females [57]. These sex-specific hypoglycemic patterns of NE input to the VMN imposed control of glycogen metabolism as hindbrain 6OHDA lesions alter hypoglycemia-associated GS and GP isoform protein expression [Briski, personal communication]. Accordingly, our work compared male versus female astrocyte responses with graded NE exposure. A key unanticipated finding of this work was the broad extent of the differential intrinsic responsiveness of male versus female primary astrocytes to NE exposure [59], which may have been a result, in part, of gonadal steroid imprinting during the critical developmental period of brain differentiation. Study outcomes documented sex differences in the noradrenergic regulation of glycogen metabolic enzyme expression that included fine versus coarse control of GS expression as well as differential sensitivity of GPbb versus GPmm to increasing NE dosages (Figure 2). NE augmented the astrocyte glycogen content over a narrow segment of the broad dose range, inferring that in the presence of glucose and absence of other crucial regulatory stimuli, NE may have a negligible impact on the net ratio of glycogen synthesis versus breakdown at most exposure levels.

Sex differences in the noradrenergic regulation of glia AR and ER expression involved dosage efficacy and direction of regulatory action. In male rats, β_1_- and β_2_-AR protein profiles exhibited bi-directional responses to increasing NE doses; female astrocytes exhibited diminished β_1_-AR content at low dose exposure but enhanced β_2_-AR expression at high NE dosages. Thus, in each sex, graded variations in noradrenergic stimulation may modulate astrocyte receptivity to NE in vivo albeit with different effects at specific signal strengths. Estradiol imposes sex-dimorphic control of hypothalamic astrocyte AMPK activity and glycogen mass through the regulation of the ER variant expression [58]. Outcomes here provide novel proof that hypothalamic astrocyte receptivity to estradiol is also controlled by NE. In the brain, ERs are stimulated by a ligand of dual origin as estradiol is both acquired from the circulation and generated locally from testosterone by aromatase action. The cellular sources(s) of neuroestradiol produced in the hypothalamus are unclear, yet both neurons and astrocytes in other neural structures express aromatase [60,61]. Current work has shown that aromatase is expressed in hypothalamic astrocytes in each sex and that this protein is inhibited by NE. NE may impose a tighter control of aromatase expression in male astrocytes as this profile was decreased in a dose-proportionate manner as opposed to a uniform noradrenergic inhibition irrespective of dosage in the female. Our findings document NE control of both the astrocyte receptivity to estradiol and the production of this ligand. Present outcomes, along with evidence that ERs govern astrocyte AR protein expression [62] and regulate NE effects on GPmm and GPbb profiles [19], reinforce the concept that astrocytes are targets for estradiol and NE signal interaction and that integration of these critical stimuli may shape stimulus-specific control of glycogen metabolism in a sex-contingent manner.

Hypothalamic astrocytes express AMPK and require catecholaminergic input for activation of this sensor during hypoglycemia [63]. In male rats, the NE regulation of glial glycogen metabolic enzyme profiles in vivo is AMPK-dependent [50]. A correlated premise of the research described above was that NE may govern astrocyte total AMPK protein expression and activity in a sex-specific manner and that such control may involve the regulation of an upstream stimulatory kinase (calcium/calmodulin-dependent protein kinase kinase-β) and an inhibitory phosphatase (protein phosphatase-1) protein expression in one or both sexes. NE was observed to cause divergent changes in astrocyte AMPK activity in male versus female glia, which likely involve upstream kinase/phosphatase-dependent versus independent mechanisms, respectively. NE elicited dose-dependent decrements in astrocyte AMPK total protein expression in each sex, yet male astrocytes were more sensitive to this suppressive action. Interestingly, noradrenergic stimulation promoted opposite changes in astrocyte pAMPK expression between the two sexes at each exposure level. In each sex, the direction of the NE effect, e.g., stimulation versus inhibition of the activated enzyme form, was reversed as the dosage levels increased in an opposite trend in each sex. AMPK monitors glycogen mass; glycogen binding to the AMPK beta subunit diminishes the phosphorylation of the enzyme’s catalytic subunit [64]. Here, astrocytes from each sex exhibited discordant astrocyte pAMPK expression and glycogen responses to NE at most exposure levels, outcomes that argue against the glycogen modulation of astrocyte sensor activity in the presence of NE. Opposite sex-specific noradrenergic regulation of pAMPK expression at individual dose levels could plausibly contribute to differential GS, GPbb and/or GPmm protein responses to those dosages but this premise remains speculative at this time. There is a credible need for further research to determine if and how NE concentration-dependent AMPK activity may affect astrocyte glycogen turnover and mass and other energy metabolic functions, namely substrate fuel uptake and catabolism in these cells. 

## 10. Conclusions and Directions for Future Research

Research discussed here bolsters growing recognition that metabolic signal-initiated patterns of hindbrain noradrenergic input impact VMN neuro-metabolic stability. VMN glycogen mass and/or turnover is a plausibly important metabolic variable that is subject to careful monitoring. As glycogen-derived substrate fuel stream has documented influence on VMN metabolic regulatory transmitter signaling, as indicated by evidence that NE causes 4CIN-reversible changes in the VMN nNOS and GAD protein expression [49], the astrocyte glycogen depot can be viewed as a plausible substrate for noradrenergic control of VMN gluco-regulatory function. There is need for consideration, however, whether glycogen-derived lactate affects VMN metabolic neurons as an energy fuel and/or signaling molecule [65]. There is high expectation that future gains in understanding of the molecular foundations of noradrenergic control of glycogen depot mass, which will require the implementation of complementary molecular, genetic and nano-technological approaches, will identify potential therapeutic targets in each sex for the neuro-protective stabilization of glycogen volume during states of systemic glucose dysregulation. Research discussed here characterizes ARs that may potentially mediate stimulus strength-specific effects of NE on glycogen metabolism. There is a clear necessity to distinguish individual AR effects and associated post-receptor signaling, e.g., calcium and cAMP, on astrocyte receptivity to NE and estradiol in vitro and in vivo, and to elucidate how NE and estradiol signals integrate to regulate glycogen-associated target proteins. The possibility that the noradrenergic regulation of glycogen metabolism may be coordinated system-wide among VMN astrocytes by means of cell-to-cell electrical communication via gap junctions is also deserving of future consideration [66]. 

Novel evidence for intrinsic sex differences in hypothalamic astrocyte responses to NE exposure in vitro provides ample justification for research efforts going forward to focus on whether intrinsic functional dimorphism in reactivity to this neurotransmitter occurs in vivo and, if so, potential causal mechanisms.

NE stimulates astrocyte synthesis of the chemokine CCL2 [67,68]. This molecule reportedly up-regulates β_2_-AR protein expression, cAMP signaling and glycogenolysis in vitro but does not coincidently modify lactate release [69]. Thus, mechanisms may exist within these glia that direct the intracellular fate of glucose liberated by glycogen in response to NE; namely, utilization in energy metabolic pathways for ATP production intended for internal use versus conversion to lactate for trafficking to neurons. The role of CCL2 in hypothalamic astrocyte receptivity to NE and the potential modulation of a NE-controlled glycogen-derived substrate fuel stream remain to be explored. 

Early reports that NE exposure in vitro only depletes a sub-fraction of cortical astrocyte glycogen content advanced the concept of stimulus-specific control of glycogen metabolism [70]. Our observations of differential noradrenergic regulation of VMN GPbb versus GPmm total protein expression in vivo advance the original prospect of hindbrain metabolic sensor mediated control of hypothalamic glycogen susceptibility to AMP- versus neurotransmitter-mediated mobilization. However, definitive proof of such control, including insight on the degree or magnitude of noradrenergic control of glycogen sensitivity to individual physiological signals, will require access to analytical tools that can discern whether this stimulus affects GP isoform activity, e.g., phosphorylation state. These findings raise critical questions as to whether GPbb and GPmm are expressed at equal or disproportionate levels within hypothalamic astrocytes in each sex, and if these isoforms act interchangeably at common target sites, or alternatively, promote disassembly of physically-distinct pools of glycogen. Under the latter circumstances, it would be informative to learn if relative masses of NE- versus AMP-sensitive glycogen in each sex are equivalent or different. It should be noted that observations of discrepant NE effects on GP isoform expression, notably GPbb protein profiles, in in vivo versus in vitro experimental models reinforce the notion that brain glycogen metabolism is evidently under concurrent control by non-adrenergic stimuli in vivo. Those findings emphasize the need for understanding of how diverse neurochemical and hormonal regulatory inputs are assimilated to enlarge or diminish the net volume of this energy reserve under specific physiological circumstances of energy homeostasis versus imbalance.

## Abbreviations

α_1_-AR	Alpha_1_-adrenergic receptor
α_2_-AR	Alpha_2_-adrenergic receptor
β_1_-AR	Beta_1_-adrenergic receptor
β_2_-AR	Beta_2_-adrenergic receptor
CaMKKβ	Calcium/calmodulin-dependent protein kinase kinase-β
ERα	Estrogen receptor-alpha
ERβ	Estrogen receptor-beta
GABA	γ-aminobutyric acid
GAD	Glutamate decarboxylase_65/67_
GPbb	Glycogen phosphorylase brain type
GPER	G-protein-coupled estrogen receptor-1
GPmm	Glycogen phosphorylase muscle type
GS	Glycogen synthase
NE	Norepinephrine
NO	Nitric oxide
nNOS	Neuronal nitric oxide synthase
PP1	Protein phosphatase-1
VMN	Ventromedial hypothalamic nucleus
